# PET Cell Tracking Using ^18^F-FLT is Not Limited by Local Reuptake of Free Radiotracer

**DOI:** 10.1038/srep44233

**Published:** 2017-03-13

**Authors:** Mark G. MacAskill, Adriana S. Tavares, Junxi Wu, Christophe Lucatelli, Joanne C. Mountford, Andrew H. Baker, David E. Newby, Patrick W. F. Hadoke

**Affiliations:** 1 University/BHF Centre for Cardiovascular Science, University of Edinburgh, Edinburgh, UK; 2 Clinical Research Imaging Centre, University of Edinburgh, Edinburgh, UK; 3 Institute of Cardiovascular and Medical Sciences, University of Glasgow, Glasgow, UK

## Abstract

Assessing the retention of cell therapies following implantation is vital and often achieved by labelling cells with 2′-[^18^F]-fluoro-2′-deoxy-D-glucose (^18^F-FDG). However, this approach is limited by local retention of cell-effluxed radiotracer. Here, in a preclinical model of critical limb ischemia, we assessed a novel method of cell tracking using 3′-deoxy-3′-L-[^18^F]-fluorothymidine (^18^F-FLT); a clinically available radiotracer which we hypothesise will result in minimal local radiotracer reuptake and allow a more accurate estimation of cell retention. Human endothelial cells (HUVECs) were incubated with ^18^F-FDG or ^18^F-FLT and cell characteristics were evaluated. Dynamic positron emission tomography (PET) images were acquired post-injection of free ^18^F-FDG/^18^F-FLT or ^18^F-FDG/^18^F-FLT-labelled HUVECs, following the surgical induction of mouse hind-limb ischemia. *In vitro*, radiotracer incorporation and efflux was similar with no effect on cell viability, function or proliferation under optimised conditions (5 MBq/mL, 60 min). Injection of free radiotracer demonstrated a faster clearance of ^18^F-FLT from the injection site vs. ^18^F-FDG (p ≤ 0.001), indicating local cellular uptake. Using ^18^F-FLT-labelling, estimation of HUVEC retention within the engraftment site 4 hr post-administration was 24.5 ± 3.2%. PET cell tracking using ^18^F-FLT labelling is an improved approach vs. ^18^F-FDG as it is not susceptible to local host cell reuptake, resulting in a more accurate estimation of cell retention.

There is a rapidly growing interest in the use of cell-based therapies in the clinical management of cardiovascular disease. These developments require complementary imaging techniques to help assess the biodistribution and retention of cells in target tissues. It is important to understand cell retention in order to understand their mechanism of action, to develop techniques which help retain cells at the site of injury and to clarify optimal dosing regimens. There are different imaging modalities which can be employed in cardiovascular cell tracking studies^1^ including; magnetic resonance imaging^2^,^3^, optical^4^, ultrasound^5^, single-photon emission computed tomography (SPECT)^6^,^7^ and positron emission tomography (PET)^8^,^9^. With PET, cells can either be directly labelled, transplanted and tracked; or indirectly tracked post-transplantation using PET reporter genes^10^. However, due to regulatory hurdles the use of reporter genes in humans is limited.

One of the most commonly applied clinical PET cell tracking approaches in cardiovascular studies uses direct cell labelling with 2′-[^18^F]-fluoro-2′-deoxy-D-glucose (^18^F-FDG)^8^,^9^,^11^,^12^,^13^,^14^,^15^,^16^. ^18^F-FDG is a glucose analogue which becomes phosphorylated and trapped intracellularly and therefore can be used as a marker of metabolic activity as well as for cell tracking. However, there are two major limitations with this technique; first ^18^F-FDG can be effluxed from labelled cells and, second, this free radiotracer can then be taken up by local host cells making analysis of cell retention difficult. These limitations have led to efforts to reduce the efflux of ^18^F-FDG from labelled cells with limited success^17^. In addition, ^18^F-FDG is rapidly taken up by inflammatory cells and has been used as a marker of inflammation^18^,^19^,^20^ and, therefore, may not be suitable for PET cell tracking in cardiovascular diseases with an inflammatory component, such as myocardial infarction, atherosclerotic plaque development and limb ischemia. With the objective of developing an improved and readily translatable PET cell tracking imaging agent which has no (or negligible) transfer to non-target cells *in vivo*, we investigated a novel approach to direct cell labelling and tracking using the thymidine analogue 3′-deoxy-3′-L-[^18^F]-fluorothymidine (^18^F-FLT). Cellular uptake of ^18^F-FLT is limited to proliferating cells during DNA synthesis^21^. Therefore, efflux of ^18^F-FLT is unlikely to be scavenged and retained by non-dividing inflammatory cells which are locally present in this model, allowing more accurate interpretation of cell movement.

As part of our efforts to translate the use of human embryonic stem cell-derived endothelial cells into the clinic for treatment of critical limb ischemia^22^, we aimed to optimise and develop methods for the accurate assessment of cell tracking post-implantation. This investigation addressed the hypothesis that any ^18^F-FLT effluxed from labelled cells would not be incorporated at the site of injection by host cells, providing an improved method for tracking endothelial cell fate in an *in vivo* model of ischemia-induced angiogenesis compared to ^18^F-FDG labelling. The key aims were to determine; 1) whether cells could be labelled with ^18^F-FLT and how this compared to ^18^F-FDG labelling, 2) the effect of radiolabelling human umbilical vein endothelial cells (HUVECs) with ^18^F-FDG and ^18^F-FLT on cell viability, proliferation and function *in vitro*, and 3) if ^18^F-FLT cell labelling is an improved approach compared with ^18^F-FDG for assessing cell fate *in vivo* due to the absence of local re-uptake of effluxed radiotracer.

## Results

### Optimisation of cell radiolabelling with ^18^F-FDG or ^18^F-FLT and characterisation of *in vitro* radiotracer efflux

**Figure 1:**
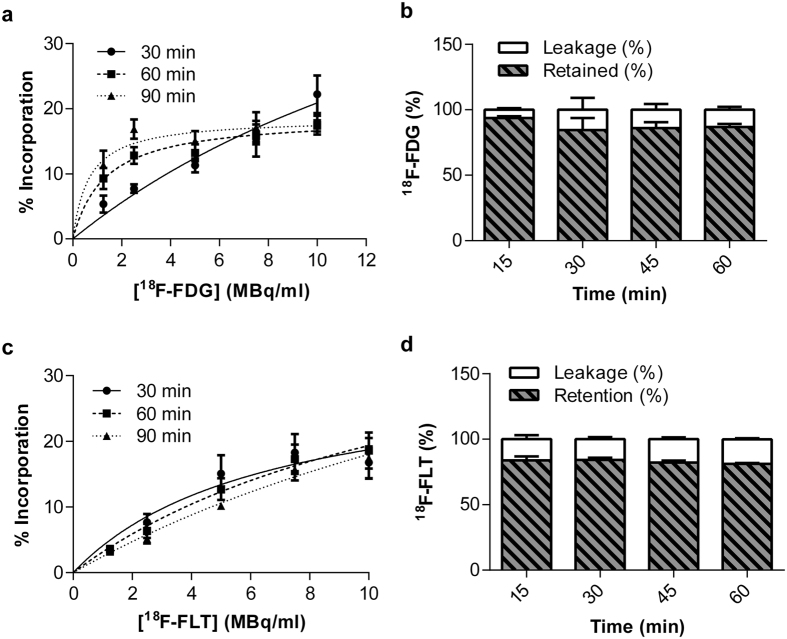
*In vitro* characterisation of radiotracer uptake and efflux from HUVECs. (**a**) Optimisation of ^18^F-FDG labelling concentrations performed at different time-points under starvation conditions (PBS), expressed as % incorporation relative to incubation medium and washes, n = 3–4. Data presented as dose-response curves for three separate incubation time-points. (**b**) Leakage of intracellular ^18^F-FDG over time at room temperature, n = 3. (**c**) Optimisation of ^18^F-FLT labelling concentrations performed at different time-points under growth conditions (EGM-2), expressed as % incorporation relative to incubation medium and washes, n = 5. Data presented as dose-response curves for three separate incubation time-points. (**d**) Leakage of intracellular ^18^F-FLT over time at room temperature, n = 4–5.

Incorporation of ^18^F-FDG into HUVECS (relative to supernatant) reached a plateau at 1.8 ± 0.1% following 90 min incubation with 5 MBq/mL in EGM-2 (Sup. Fig. 1a). When incubations were performed under starvation conditions (serum-free PBS), cellular uptake of ^18^F-FDG increased, with a plateau of 13.2 ± 1.3% reached following a 60 min incubation with 5 MBq/mL (Fig. 1a). A similar level of incorporation into HUVECs (12.7 ± 1.7%) was achieved with ^18^F-FLT following a 60 min incubation with 5 MBq/mL in EGM-2 (Fig. 1c). For both radiotracers, two PBS washes were sufficient to remove free agent from the supernatant (Sup. Fig. 1b,c).

To estimate the level of radiotracer leakage prior to administration, efflux from cells was investigated over the first hour post-labelling at room temperature. Efflux of ^18^F-FDG from cells stabilised at 13.9 ± 4.4% after 30 min (Fig. 1b). Likewise, efflux of ^18^F-FLT from the cells was stable at 17.8 ± 1.5% after 15 min (Fig. 1d).

### Assessment of the effects of radiotracer labelling on HUVEC viability, proliferation and function

**Figure 2:**
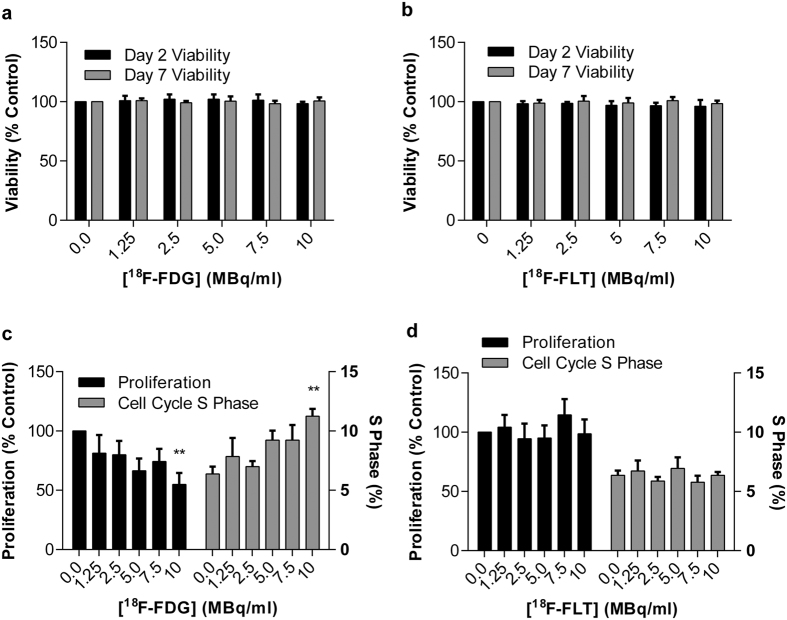
High concentrations of ^18^F-FDG (but not ^18^F-FLT), impair HUVEC proliferation. (**a**) Viability of ^18^F-FDG (n = 4–8) and (**b**) ^18^F-FLT (n = 6–7) labelled cells at 2 and 7 days post-labelling, assessed by trypan blue exclusion. The effect of (**c**) ^18^F-FDG (n = 3–6) and (**d**) ^18^F-FLT (n = 5–7) on cell proliferation 7 days post-labelling, assessed by total viable cell counts and FACs cell cycle analysis. **p ≤ 0.01 vs. control using a one-way ANOVA with post-hoc Dunnett’s test.

**Figure 3:**
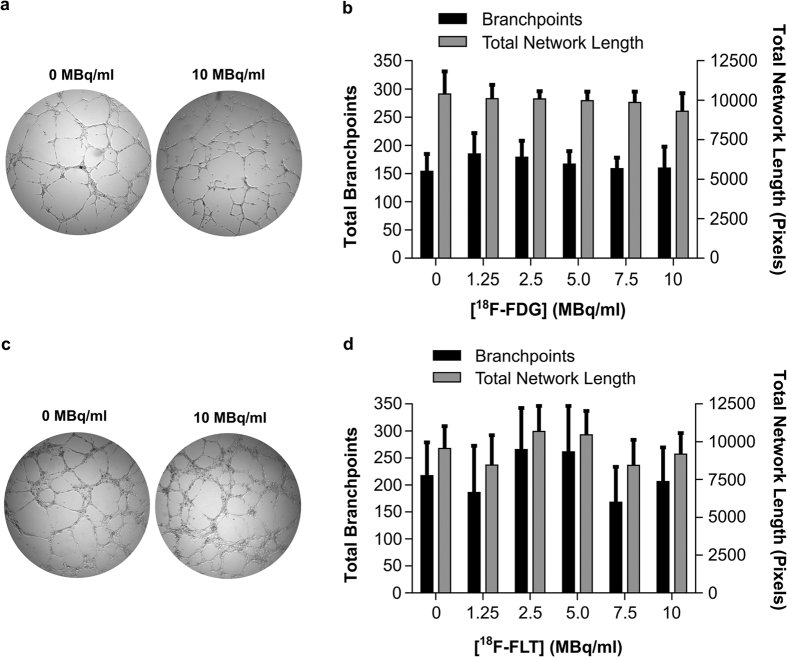
Labelling HUVECs does not alter *ex vivo* tube-like structure formation on Matrigel. (**a**) ^18^F-FDG-treated HUVEC tubule formation on matrigel matrix 7 days post-labelling and (**b**) branch-point and network length quantification, n = 4–5. (**c**) ^18^F-FLT-treated HUVEC tubule formation and (**d**) branch-point and network length quantification, n = 5–7.

Radiolabelling cells with either ^18^F-FDG or ^18^F-FLT was not associated with any alteration of cell viability (Fig. 2a,b, respectively) at the investigated concentrations. However, 7 days post-radiolabelling, HUVECs incubated with ^18^F-FDG (10 MBq/mL) showed impaired proliferation (*p* = 0.0073) vs. vehicle treated cells. ^18^F-FDG labelling (10 MBq/mL) also caused an accumulation of cells within the ‘S’ phase of the cell cycle (*p* = 0.0089, Fig. 2c) vs. vehicle treated cells, which correlated with their proliferative capacity (*p* = 0.006). No such effect was observed with ^18^F-FLT labelling (Fig. 2d). Endothelial cell function, assessed by the cells’ ability to form 2D tubule networks on Matrigel, was not altered by ^18^F-FDG or ^18^F-FLT labelling (Fig. 3).

### Dynamic PET imaging of free ^18^F-FDG and ^18^F-FLT distribution profiles

**Figure 4:**
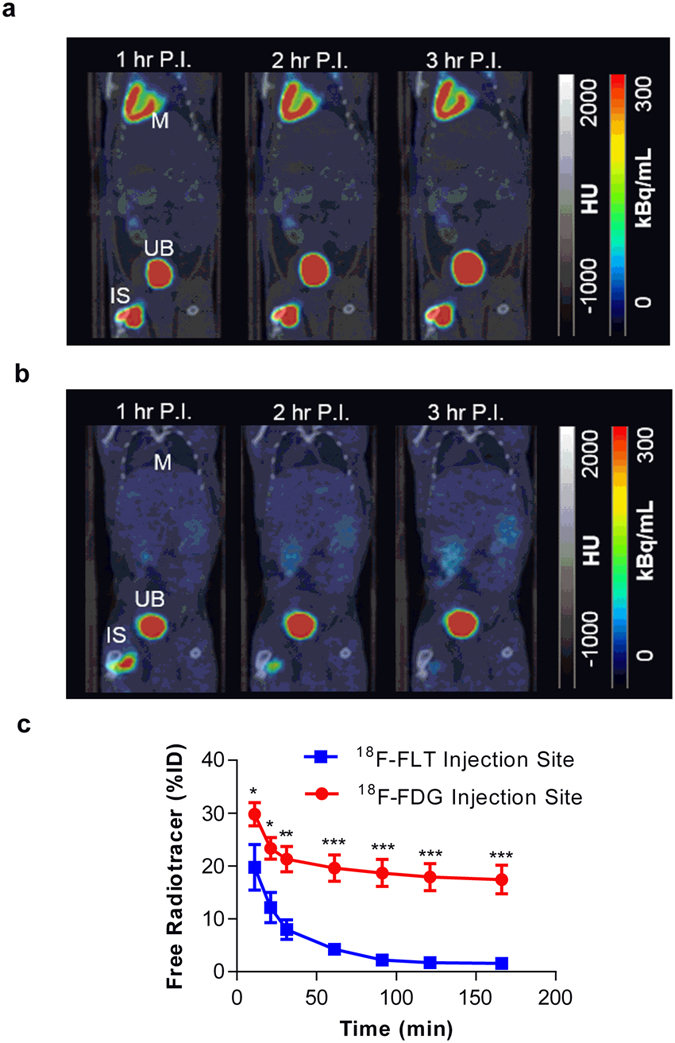
Comparison of free ^18^F-FDG and ^18^F-FLT signal profiles. Representative averaged (1 hr) images from each hour post-injection (P.I.) of (**a**) free ^18^F-FDG- and (**b**) free ^18^F-FLT. IS = injection site, M = myocardium and UB = urinary bladder. (**c**) Free ^18^F-FDG and ^18^F-FLT time activity curves at the injection site, *p < 0.05, **p ≤ 0.01, ***p ≤ 0.001 for ^18^F-FDG- vs. ^18^F-FLT, two-way ANOVA with post-hoc Bonferroni’s multiple comparisons test, n = 3.

Following injection of free ^18^F-FDG or ^18^F-FLT in mice which had undergone the induction of hind-limb ischemia, the distribution of radiotracer was dynamically imaged (Fig. 4). At the first imaging time-point (16.7 ± 2.2 min post-injection, mean ± SD, n = 6), 29.8 ± 2.1% ID and 19.8 ± 4.3% ID of ^18^F-FDG and ^18^F-FLT signals, respectively, were still present within the injection site. In experiments performed with free ^18^F-FLT, remaining radiotracer cleared completely from the injection site. In contrast, experiments performed with free ^18^F-FDG demonstrated a significantly higher signal within the injection site at all time points vs. ^18^F-FLT. At the end of the study, ^18^F-FDG failed to clear from the injection site with 17.4 ± 2.7% ID remaining (Fig. 4c). In animals which received ^18^F-FDG, radioactivity accumulated at other highly metabolic sites, namely the myocardium and brain, as well as in the kidneys and urinary bladder which is consistent with ^18^F-FDG metabolic uptake and elimination (Fig. 4a, Sup. Fig. 2a). Following injection of ^18^F-FLT, no measurable PET signal was detected in any of the major organs apart from the kidneys and the urinary bladder, consistent with known excretion route of ^18^F-FLT (Fig. 4b, Sup. Fig. 2b).

### Dynamic PET imaging of ^18^F-FDG and ^18^F-FLT-labelled HUVEC distribution profiles

**Figure 5:**
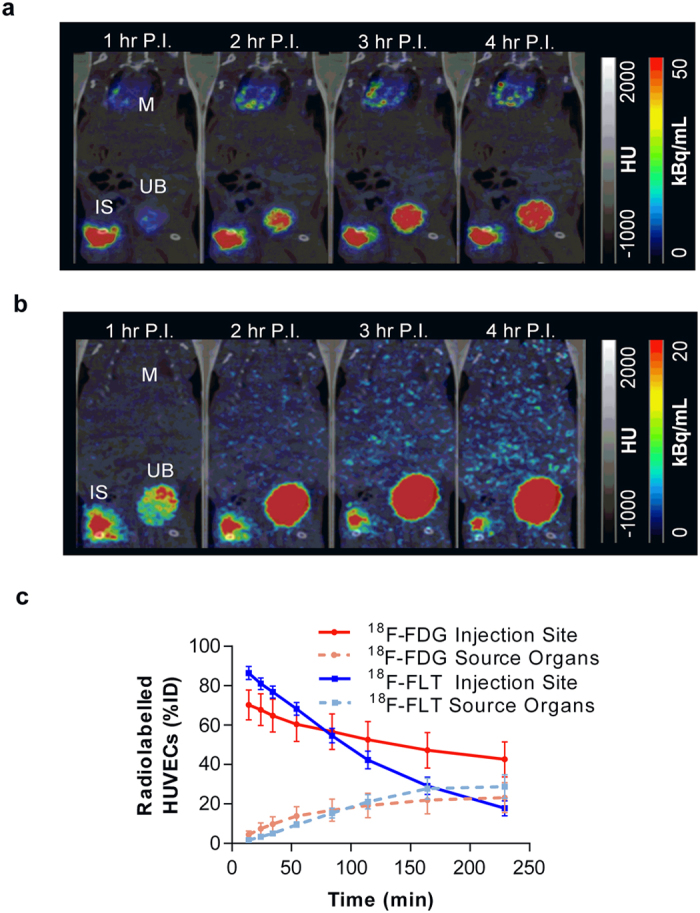
Comparison of ^18^F-FDG and ^18^F-FLT-labelled cell signal profiles. Representative averaged (1 hr) images from each hour post-injection (P.I.) of (**a**) ^18^F-FDG- and (**b**) ^18^F-FLT-labelled HUVECs. IS = injection site, M = myocardium and UB = urinary bladder. (**c**) ^18^F-FDG- and ^18^F-FLT-labelled HUVECs time activity curves at the injection site and source organs, n = 3.

In both cell labelling approaches, radioactivity in the engraftment site of mice which had undergone the induction of hind-limb ischemia reduced over the 4 hr acquisition (Fig. 5a,b). However, the rate of signal clearance with the ^18^F-FLT-HUVECs was faster compared with ^18^F-FDG-HUVECs (Fig. 5c). Similar to the free radiotracer experiment, ^18^F-FDG radioactivity accumulated in highly metabolic sites, namely the myocardium and brain, as well as in the kidneys and urinary bladder (Fig. 5a, Sup. Fig. 3a). Again, the ^18^F-FLT labelling approach did not result in uptake at any of the major organs apart from the kidneys and the urinary bladder (Fig. 5b, Sup. Fig. 3b). Neither approach resulted in accumulation of signal in the lungs, contralateral limb, liver or blood pool (Sup. Fig. 3).

**Figure 6:**
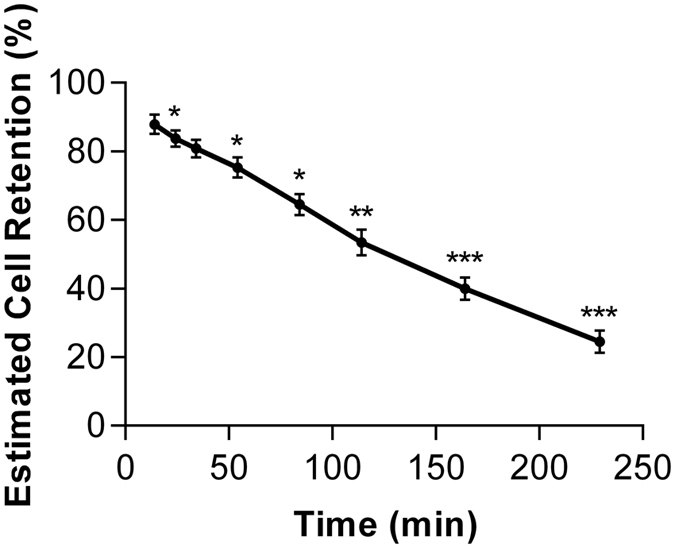
Estimated ^18^F-FLT-labelled cell retention at the engraftment site. Retention was estimated relative to the inverse efflux rate of free radiotracer based on activity uptake at the source organs, *p < 0.05, **p ≤ 0.01, ***p ≤ 0.001 vs. first time-point, one-way ANOVA with post-hoc Dunnett’s test, n = 3.

Estimation of cell retention at the engraftment site when using the ^18^F-FDG-labelling approach was hampered by local reuptake of effluxed radiotracer, as demonstrated in the free radiotracer experiments. Conversely, estimation of cell retention at the engraftment site when using the ^18^F-FLT-labelling approach was feasible; demonstrating that labelled cell retention decreased over time compared to the first time point (p ≤ 0.001 at 4 hr) (Fig. 6). At 4 hr post-transplantation, 24.5 ± 3.2% of cells remained within the engraftment site.

## Discussion

In this study, we explored the use of direct labelling of endothelial cells with ^18^F-FLT as an alternative to the commonly used ^18^F-FDG radiolabelling approach. To our knowledge, this is the first study to investigate ^18^F-FLT as a cell labelling agent. We hypothesised that, due to the nature of the ^18^F-FLT uptake mechanism, this approach would be suitable for labelling cells in culture. More importantly, we hypothesised that the ^18^F-FLT approach would not be confounded by local retention of radiotracer leakage from the transplanted cells in a murine model of critical limb ischemia; contrary to ^18^F-FDG which would be retained. We have demonstrated that both uptake and leakage of ^18^F-FLT by HUVECs was comparable to that of ^18^F-FDG. Also, ^18^F-FLT-labelling was not associated with any impact on cell characteristics; unlike ^18^F-FDG, which at high concentrations impaired the cells capacity to proliferate. In the murine model of hind-limb ischemia we demonstrated that free ^18^F-FLT is completely cleared from the injection site whereas a substantial proportion of ^18^F-FDG is retained. In ^18^F-FLT-labelled cell tracking experiments, estimation of cell retention was possible and revealed that one quarter of the cells were still present within the engraftment site 4 hours post-injection.

When comparing the level of ^18^F-FLT incorporation which was achieved in this study to other approaches, it is important to note that there are a number of factors which may affect cell uptake/yields, such as; incubation conditions, cell type, cell number, incubation volume and labelling agent uptake mechanism. Also, methods of reporting uptake/yield differ across studies making it difficult to directly compare. While uptake in this study (12%) may be considered to be at the lower end of what is reported in the literature, particularly compared to [^89^Zr]oxinate4 which has been reported to be in the range of 40–61%^23^, the levels which were achieved in this study were sufficient to accurately measure the signal within the engraftment site for up to 4 hours. In addition, studies previously performed with ^111^In-tropolone-labelling^6^,^7^ of endothelial cells have resulted in 0.1 Bq/cell, which is the same value achieved within this study. This allows for successful scaling up to clinical translation, where the total injected activity will be sufficient for *in vivo* imaging.

The negative impact of ^18^F-FDG on cell proliferative capacity is an important observation which highlights the need to assess the effect of labelling agents on cell properties prior to *in vivo* studies, an aspect which is often under-studied. Not surprisingly, we found that ^18^F-FDG-induced impairment of cell proliferation correlated with an accumulation of cells in the *S* phase of the cell cycle, indicative of radiation-induced DNA damage. The effect of ^18^F on DNA damage has been demonstrated previously^24^. Kashino *et al*. assessed the effect of poorly absorbed ^18^F ion versus highly absorbed ^18^F-FDG on double strand DNA breaks. Intracellular ^18^F in the form of FDG caused more double stranded breaks and lower cell proliferation in Chinese Hamster Ovary cells compared with ^18^F ion. Similarly, ^18^F-FDG labelling of adipose-derived stem cells also caused DNA damage and impaired proliferation^25^. In contrast, however, other studies using mesenchymal and embryonic stem cells have reported no such detrimental effects following labelling with ^18^F-FDG^15^,^26^. These conflicting reports may be due to differences in uptake levels which were achieved across the studies. Data from our study are consistent with prior observations that cellular damage by ^18^F radiolabelling is dependent on the radiotracer used as this influences the delivery of dose to key intracellular structures. The lack of detrimental effect evident on the total population of HUVECs post-^18^F-FLT may be due to this approach only labelling a subset of the cells which are undergoing S-phase of the cell cycle due to the uptake mechanism of this radiotracer^21^,^27^. The uptake mechanism of ^18^F-FDG^28^ is likely to result in greater homogeneity of individual cell uptake, making total population effects more obvious.

In order to accurately quantify cell tracking, an ideal cell-labelling agent should be specific for the transplanted cell with no or negligible transfer to non-target cells locally or systemically *in vivo*. This allows for an accurate estimation of cell engraftment and distribution^29^. Efflux of labelling agents from cells can interfere with this goal. In this study we demonstrated that prior to injection, around 14–18% of the labelling agent had leaked from the cells *in vitro*. This degree of leakage is similar to other reported values when using direct cell labelling methods^25^, albeit some studies have reported cell efflux values as high as 40–50% up to 2 hours post-labelling^15^,^17^. Differences in radiotracer leakage are likely due to different levels of incorporation which were achieved across studies and is also likely to be cell-type dependent. As a degree of labelling agent leakage from cells is unavoidable when using direct cell labelling methods, consideration of the specificity of the radiotracer uptake mechanism is paramount to best tailor *in vivo* cell tracking techniques to a given application, particularly in studies with a highly localised delivery site. In light of the well described uptake mechanisms of ^18^F-FDG and ^18^F-FLT^21^,^28^, we hypothesised that efflux of ^18^F-FDG would result in local retention unlike ^18^F-FLT which would be cleared. We were led to this hypothesis due to a number of factors. Activation and infiltration of inflammatory cells to the injury site is a major event during hind-limb ischemia^30^, and results in a local increase of highly metabolically-active cells. In other murine models of cardiovascular injury, acute recruitment of inflammatory cells to an injury site can occur in the first few hours of reperfusion (1–5 hr)^31^,^32^. Another source of local radiotracer retention may include increased metabolism during skeletal muscle ischemia^33^, as well as locally increased energy demand due to ischemia-induced switching of native endothelial cells from a quiescent to an angiogenic phenotype^34^,^35^.

In this current study, the leakage profiles measured were similar for both radiotracers. In addition, the finding that a significant proportion of ^18^F-FDG is retained within the ischemic hind-limb indicates that the signal measured in the injection site when using ^18^F-FDG labelling represents three different compartments. Specifically: ^18^F-FDG-labelled HUVECs, secondary cell labelling via transfer of ^18^F-FDG to local cells, and free ^18^F-FDG. Conversely, using the ^18^F-FLT approach, there are two major compartments: ^18^F-FLT-labelled cells and free ^18^F-FLT. A third potential minor compartment would include free or metabolised (in various cells) radiotracer, although this is unlikely based on the results collected during the free radiotracer experiments. Therefore, ^18^F-FLT-labelling of cells allows for more accurate assessment of cellular engraftment/kinetics within highly metabolic sites compared with ^18^F-FDG based approaches. This ^18^F-FLT-based cell tracking approach will also be particularly valuable in cell tracking studies within the heart (such as following myocardial infarction), given that free ^18^F-FLT is not taken up by cells in the myocardium. The fact that ^18^F-FDG is often used in studies of cardiac inflammation and injury further indicates that ^18^F-FDG is not optimal for these purposes^19^,^36^. Therefore, the novel ^18^F-FLT cell labelling approach presented here can be valuable in multiple clinical cardiovascular applications and potentially cell tracking in other biomedical applications.

Due to improved compartmental definition within the ^18^F-FLT signal of this study, an attempt has been made to estimate cell retention within the engraftment site. This interpretation is based on the inverse rate of free radiotracer accumulation relative to the total injected dose within source organs, which for ^18^F-FLT consisted of the elimination organs (kidney and bladder). It should be noted that this interpretation may be confounded by variability in urinary excretion that can affect uptake at the elimination organs. However, urinary voiding was not observed during these scanning sessions, and care was taken to ensure consistent animal preparation and surgical procedures across individual animals. Within this study, estimated cell retention at the engraftment site was 24.5 ± 3.2% at 4 hours post-injection, suggesting a rapid clearance of cells. This estimation follows the range of early retention rates reported in previous clinical studies^37^,^38^,^39^ using alternative cell tracking methods, with one study in particular reporting an average cell retention rate of 21.3 ± 5.2% at 2 hours post-injection following transendocardial delivery of bone marrow mononuclear cells^40^. Thus, despite cell retention post-injection being largely dependent on the administration route, type of cells and type of pathology; the rates reported in this study are in-line with previously reported values in other applications. In addition to cell retention, this study explored the potential fate of ^18^F-FLT-labelled cells which may have left the engraftment site by evaluating the signals within the liver and lungs. These organs demonstrated negligible levels of radioactivity, indicating negligible accumulation of cells within these structures. In contrast, the previously demonstrated fate of intravenously administered ^18^F-FDG-labelled mesenchymal stem cells demonstrated significant accumulation within these organs^26^. It is conceivable that the majority of the injected cells in this study have lost their cellular arrangement or died locally and released free radiotracer, which could explain the observed high uptake in the kidneys and urinary bladder. This is consistent with prior reports that only 1–5% of delivered cells engraft within the target site for regeneration^41^.

While the lower impact of ^18^F-FLT-labelling on the total population of HUVECs is certainly an advantage over ^18^F-FDG-labelling endothelial cells, labelling only a subpopulation of cells has some limitations. In studies which administer smaller cell doses, the lack of whole population labelling may limit the ability to detect small or diffuse cell populations. In addition, heterogeneous cell uptake could further complicate interpretation of the cell retention profile when using direct cell labelling methods. Cells which have taken up larger amounts of radioactivity could be at greater risk of damage, and this may affect their fate following transplantation (thus complicating interpretation of cell fate). However, in this particular short-term cell tracking study this effect is likely to be less prominent than in long-term tracking. Moreover, in this study we have demonstrated that radiolabelling of cells with ^18^F-FLT had no effect on cell viability, proliferation and function *in vitro*. It should be noted that the value of this novel approach, as with any other ^18^F-based stem cell labelling and tracking approach, lies on the early assessment of cell engraftment and biodistribution within the first few hours post-transplantation. For studies requiring longer term cell tracking other imaging approaches using PET tracers with longer lived radioisotopes such as ^89^Zr-Oxinate4^23^ and ^64^Cu–PTSM^42^ are valuable alternatives, in addition to the widely used ^111^In-tropolone or ^99m^Tc-HMPAO labelling for SPECT cell tracking^6^,^7^,^43^. Alternatively, magnetic resonance imaging of iron nanoparticle labelled cells has also been shown to be a suitable approach for longitudinal cell tracking^2^.

## Conclusion

A novel methodology for labelling human endothelial cells using ^18^F-FLT was shown to result in efficient labelling of cells without affecting cell characteristics and, more importantly, without resulting in local retention of effluxed ^18^F-FLT from the labelled HUVECs. Consequently, cell labelling with ^18^F-FLT for *in vivo* tracking using PET is an improved technique to evaluate cell engraftment/kinetics at the delivery site. ^18^F-FLT direct cell labelling is a fully translatable method and could be used to assess cell engraftment during stem cell therapy in both preclinical and clinical studies.

## Methods

### Radiotracers

^18^F-FDG was prepared using standard FASTlab FDG cassettes (GE Healthcare, UK) or TRACERlab MX kits (ROTEM, Israel) and formulated in phosphate buffer solution. ^18^F-FLT was prepared using a standard FASTlab FLT Cassette (GE Healthcare, UK) and was formulated in 9% ethanol in water. Radiochemical purity was >99% for both ^18^F-FDG and ^18^F-FLT.

### Cell Culture

In this study, HUVECs were chosen as a model endothelial cell line as they share many characteristics with other pro-angiogenic endothelial cell therapies^44^. Pooled donor primary HUVECs (C-12208, PromoCell, Germany) were cultured in endothelial growth medium 2 (EGM-2) (PromoCell, Germany) on 0.1% gelatine (Sigma, USA)-coated culture flasks/plates and, where stated, Dulbecco’s Phosphate-Buffered Saline (DPBS) (Lonza, Switzerland) or endothelial basal medium (EBM) (PromoCell, Germany). HUVECs were passaged when confluent using 0.05% trypsin/EDTA (Gibco, UK) and used between passages 2–8.

### Cell Radiotracer Labelling Optimisation

HUVECs were seeded at 0.25 × 10^6^ cells/well in a 6-well plate and left to adhere overnight. To yield maximal incorporation of radiotracer in cells, different incubation mediums were investigated based on known radiotracer uptake mechanisms^21^,^28^. As ^18^F-FDG is a glucose analogue, uptake was assessed both in the presence of full growth medium (EGM-2) and under starvation (serum free PBS) conditions. ^18^F-FLT uptake was assessed using full growth medium (EGM-2). Immediately prior to incubation, adhered cells were washed with DPBS, then 1 mL of the radiotracer solution/well (1.25–10 MBq/mL) was added. The cells were then returned to the incubator (37 °C, 5% CO_2_, 100% humidity) for 30, 60 or 90 min. At the end of the incubation each well was washed twice with 1 mL DPBS, with the incubation medium and washes collected separately for counting. The remaining cells were then lysed by the addition of 200 μL RIPA buffer and transferred to corresponding tubes. The activity of each sample was measured using an automatic gamma counter (Wizard 1470 Gamma Counter, Perkin Elmer), and intracellular uptake was calculated as a percentage of the total yield from incubation medium and washes. Optimised labelling conditions were used for *in vivo* imaging studies by linear “scaling-up” of volume, total activity and cell density (2 × 10^6^ cells).

### Cell Radiotracer Leakage

To assess *in vitro* cellular radiotracer leakage prior to injection, 0.25 × 10^6^ radiolabelled cells were suspended in 60 μL of EBM and kept at room temperature for up to 1 hour. Cells were then re-spun (200 × g, 5 min) at selected time intervals. The supernatant was collected in a separate vial, after which the remaining pellet was lysed by the addition of 200 μL RIPA buffer (Sigma, USA). Radioactivity was then measured as described in the previous section.

### Cell Viability and Proliferation

Following radiolabelling with ^18^F-FDG or ^18^F-FLT, cells were washed and returned to EGM-2. Two days post-radiolabelling, cell viability was assessed by trypan blue exclusion assay. HUVECs were trypsinised, spun and aliquoted before adding 10% trypan blue solution to a final concentration of 0.04% (Bio-Rad, USA). Using a hemocytometer, viable cells were identified and counted by the absence of blue dye uptake. HUVECs were then reseeded into a 6-well plate at 0.05 × 10^6^ and cultured for a further 5 days in EGM-2 with one medium change. Cell viability was calculated again, and proliferation was calculated using the total number of viable cells. Results were normalised to a vehicle control (1 hr PBS for ^18^F-FDG experiments, 1 hr EGM-2 for ^18^F-FLT experiments) which was washed and returned to EGM-2 in parallel with radiolabelled groups.

### FACs Cell Cycle Analysis

Cell cycle analysis was carried out seven days post-labelling using cells re-plated at 0.1 × 10^6^ cells/well on day 2. Briefly, cells were collected and fixed in 70% ethanol at 4 °C for at least 1 hr. The fixed cells were then spun and re-suspended in 250 μL PBS containing 50 μg/mL Ribonuclease A and incubated for 1 hr at 37 °C. Lastly, 40 μg/mL of propidium iodide was added to each sample before analysis by flow cytometry. Results were compared to a vehicle control (1 hr PBS for ^18^F-FDG experiments, 1 hr EGM-2 for ^18^F-FLT experiments) which was washed and returned to EGM-2 in parallel with radiolabelled groups.

### Tubule Formation Assay

Endothelial cell function was assessed seven days post-radiolabelling using a Matrigel (Corning, USA) tubule formation assay, as previously described^45^,^46^. Briefly, 1 × 10^4^ cells were seeded onto Matrigel-coated 96 well plates (50 μL Matrigel/well) in EGM-2. After 6 hours at 37 °C (5% CO_2_, 100% humidity), capillary-like endothelial cell networks were examined by phase contrast microscopy (×5 lens). The angiogenesis analyser plugin for image J^47^ was used to quantify the capacity of the cells to form tubule networks. Results were compared to a vehicle control (1 hr PBS for ^18^F-FDG experiments, 1 hr EGM-2 for ^18^F-FLT experiments) which was washed and returned to EGM-2 in parallel with radiolabelled groups.

### Murine Hind-Limb Ischemia Model and Free Radiotracer/Radiolabelled Cell Administration

Experimental procedures were approved by the local University of Edinburgh animal ethics committee, and were authorized by the Home Office under the Animals (Scientific Procedures) Act 1986. Male CD1-Foxn1nu mice aged between 12–18 weeks were used in this study. Anaesthesia was induced and maintained using isoflurane (1.5%, Oxygen 1 L/min) during the surgical procedure. The hind-limb ischemia was induced as described^48^. Briefly, the left femoral artery was ligated and cut proximal to the epigastric branch, and the saphenous artery was ligated distal to the popliteal branch. Post-surgery, mice received an intramuscular injection of free ^18^F-FDG or ^18^F-FLT (500–900 kBq), or ^18^F-FDG or ^18^F-FLT-labelled cells (100–800 kBq) into the ischemic limb. Free radiotracer (diluted in 15 μL EBM-2) or labelled HUVECs (1 × 10^6^ diluted in 15 μL EBM-2) were injected into three sites (5 μL/site) along the projection of the adductor muscle. The wound was closed with interrupted sutures (4/0 silk) and the animal placed into the nanoPET/CT scanner. Anaesthesia was maintained throughout the imaging session using isoflurane (1.5%, 0.5:0.5 Oxygen/Nitrous Oxide, 1 L/min).

### PET/CT Acquisition, Reconstruction and Image Analysis

All PET data were acquired using a nanoPET/CT scanner (Mediso, Hungary). Post administration of free ^18^F-FDG/FLT or radiolabelled cells, a 240 min whole-body emission scan was obtained using a 1:5 coincidence mode. Then, a CT scan was acquired (semi-circular full trajectory, maximum field of view, 480 projections, 35 kVp, 400 ms and 1:4 binning) for attenuation correction. PET data was reconstructed into 3 × 10, 3 × 30 and 2 × 60 min frames using Mediso’s iterative Tera-Tomo 3D reconstruction algorithm and the following settings: 4 iterations, 6 subsets, full detector model, normal regularisation, spike filter on, voxel size 0.6 mm and 400–600 keV energy window. PET data were corrected for randoms, scatter and attenuation.

Reconstructed scans were imported into PMOD 3.4 software (PMOD Technologies, Switzerland) and volumes of interest (VOIs) were drawn around organs of interest and sites displaying a higher radioactivity concentration than background. Source organs for ^18^F-FDG included the myocardium, brain, kidneys and bladder; and for ^18^F-FLT included the kidneys and bladder. Radioactivity in the blood was estimated using the blood pool in the left ventricular cavity. At each time point, the measured activity at different sites was expressed as the percent injected dose (%ID). The percentage cell retention within the engraftment site (IM) at each time-point was estimated relative to the inverse efflux rate of free radiotracer, based on activity uptake at the source organs (SO). This calculation assumes that the rate of clearance (free radiotracer and radiotracer released form lysed cells) can be estimated as total activity (100% injected dose) minus source organ activity.

### Statistical Analysis

The following statistical analyses were carried out using GraphPad Prism V.6, as detailed in figure legends; two-way ANOVA with post-hoc Bonferroni’s multiple comparisons test, one-way ANOVA with post-hoc Dunnett’s test and correlation analysis. A two-sided *p* value of <0.05 was considered statistically significant. GraphPad prism was also used to generate all graphs, representative of the mean ± SEM.

## Additional Information

**How to cite this article:** MacAskill, M. G. *et al*. PET Cell Tracking Using ^18^F-FLT is Not Limited by Local Reuptake of Free Radiotracer. *Sci. Rep.* **7**, 44233; doi: 10.1038/srep44233 (2017).

**Publisher's note:** Springer Nature remains neutral with regard to jurisdictional claims in published maps and institutional affiliations.

## Supplementary Material

Supplementary Information
