# Supplementary material for: PET Cell Tracking Using 18F-FLT is Not Limited by Local Reuptake of Free Radiotracer
Source: Sci Rep. 2017 Mar 13;7:44233. doi: 10.1038/srep44233 (PMC5347009; doi:10.1038/srep44233)
Supplement: Supplementary Information [file srep44233-s1.doc]

**PET Cell Tracking Using ^18^F-FLT is Not Limited by Local Reuptake of Free Radiotracer**

**Supplementary**

Mark G. MacAskill^1^*, Adriana S. Tavares^1^, Junxi Wu^1^, Christophe Lucatelli^2^, Joanne C. Mountford^3^, Andrew H. Baker^1^, David E. Newby^1^, Patrick W.F. Hadoke^1^.

1. University/ BHF Centre for Cardiovascular Science, University of Edinburgh, Edinburgh, UK
2. Clinical Research Imaging Centre, University of Edinburgh, Edinburgh, UK
3. Institute of Cardiovascular and Medical Sciences, University of Glasgow, Glasgow, UK

**a**

**b**

**c**

**Supplementary Figure 1. Optimisation of radiotracer labelling conditions and washing efficiency. a)** Assessment of ^18^F-FDG uptake in the presence of full growth medium at various concentrations and incubation times, n=3-4. **b)** Demonstration of the efficacy of PBS washes to remove excess, unbound ^18^F-FDG (n=3) and **c)** ^18^F-FLT (n=5).

b

a

**Supplementary Figure 2. Time activity curves following injection of free radiotracer demonstrating accumulation of signal within source organs, but not at other sites of interest. a)** ^18^F-FDG signal distribution over the first 3 hr post-injection, n=3. **b)** ^18^F-FLT signal distribution over the first 3 hr post-injection, n=3.

**b**

**a**

**Supplementary Figure 3. Time activity curves following injection of radiolabelled-HUVECs demonstrating accumulation of signal within source organs, but not at other sites of interest. a)** ^18^F-FDG signal distribution over the first 4 hr post-injection, n=3. **b)** ^18^F-FLT signal distribution over the first 4 hr post-injection, n=3.
